# Progress and Persistent Disparities in Patient Access to Electronic Health Information

**DOI:** 10.1001/jamahealthforum.2023.3883

**Published:** 2023-11-10

**Authors:** Chelsea Richwine

**Affiliations:** 1 Office of the National Coordinator for Health Information Technology, Washington, DC

## Abstract

**Importance:**

Patient access to electronic health information (EHI) available via online medical records and through patient portals has been shown to help individuals make informed health decisions, which are associated with better health outcomes.

**Objective:**

To assess progress in patient engagement with EHI and to identify racial or ethnic disparities in access to patient portals.

**Design, Setting, and Participants:**

This was a repeated cross-sectional study using data from the US Health Information National Trends Survey (HINTS), a nationally representative survey of US adults that tracks individuals’ access and use of their health information. Six cycles of HINTS were included (2014, 2017-2020, 2022); data for the disparities analysis came from the 2022 HINTS. Data analyses were performed in April 2023.

**Main Outcomes and Measures:**

Patient reports of (1) being offered portal access by a health care provider (HCP); (2) being encouraged by the HCP to use the portal; (3) accessing their portal; and (4) using the portal for various purposes. Additional key measures included methods used to access portals and self-reported ease of understanding information contained in the online medical records or portals.

**Results:**

The total study population included 22 266 individuals (mean [SE] age, 49.9 [0.15] years) of whom 13 348 (54%) were female; 909 (5%) self-identified as Asian, 3523 (12%) as Black, 3178 (14%) as Hispanic, 13 555 (66%) as White, and 785 (3%) as another or more than 1 race. Nationally, patient portal access increased each year from 2014 through 2022, with a 46% increase observed between 2020 (n = 3319) and 2022 (n = 5437). However, in 2022, Black and Hispanic individuals reported being offered access to a portal by their HCP at significantly lower rates compared with White individuals (73% vs 81%; χ^2^_1_ = 22.24; *P* < .001; and 62% vs 81%; χ^2^_1_ = 135.57; *P* < .001, respectively) as well as accessing a patient portal at lower rates (60% vs 70%; χ^2^_1_ = 23.80; *P* < .001; and 57% vs 70%; χ^2^_1_ = 49.02; *P* < .001, respectively).

**Conclusions and Relevance:**

The findings of this repeated cross-sectional study of US adult respondents to the HINTS suggest that access to and engagement with patient portals increased significantly from 2014 through 2022, but racial and ethnic disparities in patient access persisted in 2022. However, there were no significant differences in use or understanding of information available in the online medical records among those who accessed them, which suggests that efforts to promote equitable opportunities to access EHI would likely be associated with increased patient access.

## Key Points

**Question:**

How did patient access to electronic health information via online medical records and patient portals change during the COVID-19 pandemic?

**Findings:**

This repeated cross-sectional study of 22 266 US adults using data from 6 cycles of the Health Information National Trends Survey (2014, 2017-2020, 2022) found that patient access to electronic health information (EHI) accelerated during the pandemic, but racial and ethnic disparities persisted. There were no disparities found in use or understanding of EHI among those who accessed it.

**Meaning:**

The findings of this repeated cross-sectional study indicate that overall, patient access to EHI has improved; however, efforts are needed to promote equitable opportunities by encouraging all patients to access and use their portals.

## Teaser

This repeated cross-sectional study evaluates trends in patient engagement with electronic health information before and after the COVID-19 pandemic and identifies racial and ethnic disparities in access to patient portals.

## Introduction

A silver lining of the COVID-19 pandemic was the acceleration of patient access to electronic health information (EHI) in online medical records and patient portals, which has been shown to help patients make informed decisions about their health and has been associated with better health outcomes.^1,2^ Patient portal access has been on the rise for nearly a decade given the federal efforts to empower individuals to make informed decisions about their health by increasing patient access to EHI.^3^ In 2020, the Office of the National Coordinator for Health Information Technology published the Cures Act Final Rule (Cures Rule),^4^ which called on health information technology (IT) developers to adopt secure, standards-based application programming interfaces (APIs) that could enable patients to access their EHI using health apps via a smartphone or other methods, and prohibited health care professionals, developers, and other relevant actors from interfering with an individual’s right to access their own records.^5,6^ These anti-information blocking regulations play an important role in affirming patient access to EHI, which is central to meeting patient demand for getting timely access to test results and clinical notes.^7,8^

After several years of steady growth, patient access increased sharply in 2020 through 2022. This may be attributed to greater reliance on telehealth visits during the COVID-19 pandemic,^9,10,11^ which likely increased demand for portal access to send messages to health care professionals, view COVID-19 test results, and download or transmit information.^12^ The growth in patient access may stem from an increase in practitioners offering access to patient portals, which enables patients to access their online medical records, and encouraging their use. Furthermore, app-based access to EHI increased during the same period, whereas web-only access decreased, suggesting that patients had greater means of accessing their EHI because the health care industry began implementation of the Cures Rule.

Despite substantial progress in patient access during the past 8 years, growth has not occurred equitably. Historically, Black and Hispanic patients have been less likely to report being offered patient portal access by an HCP compared with White individuals,^13,14^ which has negative downstream implications for access^15^ and use of EHI via patient portals as well as third-party health apps, which both rely on portal credentials for authorization. Evaluating differences in rates of patients being offered the opportunity to access a portal and being encouraged to use it is critical to assessing progress toward equitable access to EHI and speaks to the importance of practitioners in enabling access to online medical records via patient portals.

The overarching goal of this study was to assess progress toward equitable patient access to EHI 2 years after the start of the COVID-19 pandemic, and to identify persistent racial and ethnic disparities. First, this study identified trends in patient reports of being offered a portal and accessing it between 2014 and 2022, and identifies changes in methods of access between 2020 and 2022. Second, to understand whether patients have equitable opportunities to access their EHI, this study identified racial and ethnic disparities in patient reports of being offered and encouraged by their practitioners to use patient portals in 2022, as well as differences in patient-reported access, use, and understanding of information contained in portals.

## Methods

This repeated cross-sectional study did not require approval or consent from an ethics committee because it used only publicly available data and did not involve human participants. We followed the Strengthening the Reporting of Observational Studies in Epidemiology (STROBE) reporting guideline for cross-sectional studies.

### Data Collection and Study Population

Data for this repeated cross-sectional study came from the Health Information National Trends Survey (HINTS), a nationally representative survey of US adults that tracks individuals’ access and use of their health information. The sample was restricted to respondents who had a health care visit in the past 12 months, and thus, had a reason to access their online medical record. eTable 1 in Supplement 1 provides details on sample size and response rates for each survey year used in the analyses and additional details regarding the HINTS survey design and weighting.^16^

Trends in patient access were analyzed across 6 cycles of the HINTS, from 2014 to 2022 (N = 22 266). Disparities in patient access were analyzed across the 2 most recent cycles of the HINTS (n = 3319 in 2020; n = 5437 in 2022).

### Measures and Outcomes

Main outcomes for patient engagement with their online medical records via patient portals and health apps included patient reports of (1) being offered online access to their medical records by a health care provider (HCP; specifically, an individual health care professional); (2) being encouraged by any of their HCPs to use their online medical record or patient portal; (3) accessing their online medical records or patient portal at least once in the past year; and (4) using their online medical records or patient portal to view, download, or send information (eTable 2 in Supplement 1 lists the survey questions). Outcomes were assessed over time and by respondent-reported race and/or ethnicity. Analyses were conducted for 3 mutually exclusive racial and ethnic groups: Non-Hispanic Black or African American; Hispanic, Latine, or of Spanish origin; and Non-Hispanic White (hereafter, Black, Hispanic, and White).

The analyses used White as the reference group because prior literature has established disparities in patient engagement outcomes for Black and Hispanic individuals relative to White individuals, and an objective of this study was to determine whether these disparities persist in 2022. We did not focus on those identifying as Non-Hispanic Asian, other race, or multiple races because, to our knowledge, no previous literature has identified access-related disparities for these groups.

Additionally, we evaluated the methods (ie, via app, website, or both) used to access online medical records. Among those who accessed their online medical record during the past year, we assessed their self-reported ease of understanding the information made available through it.

### Statistical Analysis

Differences in outcomes between years and across racial and ethnic groups were assessed using χ^2^ tests of independence. All analyses used survey weighting procedures with jackknife replicate weights to account for the complex survey design. Statistical tests were 2-tailed and *P* values < .05 were considered statistically significant. Data analyses were performed in April 2023 using Stata/SE, version 15.1 (StataCorp).

## Results

The total study population included 22 266 patients (mean [SE] age, 49.90 [0.15] years) of whom 8531 (46%) identified as male and 13 348 (54%) identified as female. The race and ethnicity of the participants were as follows: 909 (5%) self-identified as Asian, 3523 (12%) as Black; 3178 (14%) as Hispanic; 13 555 (66%) as White; and 785 (3%) as another race or more than 1 race (including American Indian/Alaska Native or Native Hawaiian/other Pacific Islander).

### Trends in Patient Access

**Figure 1.**
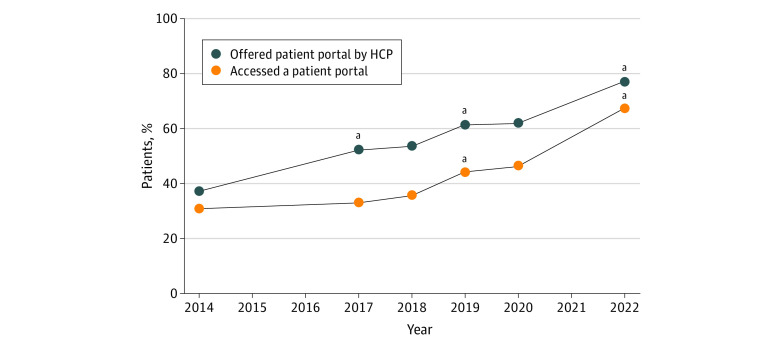
Percentage of Total Sample of US Health Information National Trends Survey (HINTS) Respondents Who Were Offered Access to a Patient Portal and Who Accessed It, 2014 to 2022 Offered a patient portal indicates % of individuals who reported being offered access to online medical records or a patient portal by an HCP. Accessed a patient portal indicates % of individuals who reported they accessed their portal at least once in the past 12 months. Only includes individuals who had a health care visit in the past 12 months. Data collected from HINTS 4, cycle 4 (2014); HINTS 5, cycles 1-4 (2017-2020); and HINTS 6 (2022). HCP indicates a health care provider (ie, an individual practitioner). ^a^Statistically significant increase from prior year (*P* < .05). Supporting data are available in eTable 3 in Supplement 1.

**Figure 2.**
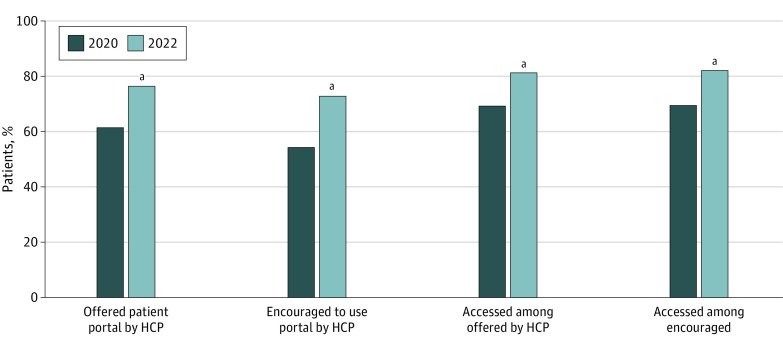
Patient Reports of Being Offered or Encouraged to Use a Portal by an HCP and Patient-Reported Portal Access (Overall and Among Those Who Were Offered or Encouraged) Offered patient portal by HCP indicates % of individuals who reported being offered access to online medical records or a patient portal by an HCP. Encouraged to use portal by HCP indicates % of individuals who reported they were encouraged by HCPs to use a patient portal. Accessed among offered (encouraged) indicates % of individuals who reported they accessed their portal at least once in the past 12 months, from among those who were offered (encouraged). These data include only individuals who had a health care visit in the past 12 months. Data collected from HINTS 5, cycle 4 (2020) and HINTS 6 (2022). HCP indicates a health care provider (ie, an individual practitioner). ^a^Statistically significant increase from prior year (*P* < .05). Supporting data are available in eTable 4 in Supplement 1.

The share of individuals who were offered and accessed a patient portal increased each year from 2014 through 2022 (Figure 1; eTable 3 in Supplement 1), with marked growth observed between 2020 and 2022. There was a 46% increase in the share of individuals who reported accessing their patient portal in the past 12 months (compared with a 5% increase in 2019 through 2020). Growth in portal access was accompanied by a 24% increase in the share of individuals who reported being offered access to a patient portal by their HCP in 2020 through 2022 (62% vs 77%; χ^2^_1_ = 218.15; *P* < .001), as well as a 34% increase in the share of individuals who reported being encouraged by their HCP to use the patient portal (55% vs 73%; χ^2^_1_ = 319.49; *P* < .001) (Figure 2; eTable 4 in Supplement 1). Portal access also increased in 2020 through 2022 among individuals who were offered access to a portal (70% vs 81%; χ^2^_1_ = 110.70; *P* < .001) and who were encouraged to use it (70% vs 83%; χ^2^_1_ = 125.21; *P* < .001).

**Figure 3.**
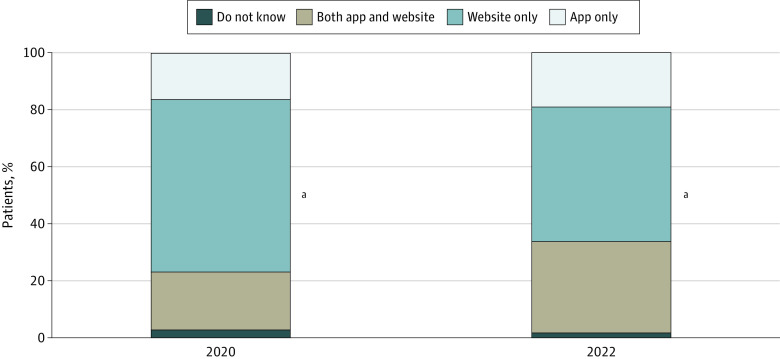
Methods of Accessing Online Medical Records or Patient Portal, 2020 to 2022 Only includes individuals who accessed their online medical record and had a health care visit in the past 12 months. Data collected from US Health Information National Trends Survey (HINTS) 5, cycle 4 (2020); and HINTS 6 (2022). ^a^Statistically significant increase from prior year (*P* < .05). Supporting data available in eTable 5 in Supplement 1.

App-based access to EHI also increased in 2020 through 2022 (Figure 3; eTable 5 in Supplement 1). There was a 57% increase in the share of individuals who accessed their online medical record or patient portal via both an app and a website (21% vs 32%; χ^2^_1 _= 78.46; *P* < .001), and a 22% decrease in web-only access (60% vs 47%; χ^2^_1_ = 78.63; *P* < .001).

### Disparities in Patient Access

In 2022, the race and ethnicity of participants (mean [SE], 49.4 [0.23] years; 3160 [53%] female and 1938 [46%] male [<1% other]) were as follows: 253 (5%) self-identified as Asian, 936 (12%) as Black, 908 (15%) as Hispanic, 3159 (64%) as White, and 181 (4%) as another or more than 1 race (including American Indian/Alaska Native or Native Hawaiian/other Pacific Islander). Most of the sample was college educated (4145 [73%]), located in an urban area (4718 [88%]), covered by health insurance (5080 [93%]), and had internet access (4581 [88%]). The sample was balanced across 4 adult age groups: 18 to 34 years (769 [23%]), 35 to 46 years (870 [20%]), 47 to 64 years (1781 [33%]), and 65 years or older (2017 [23%]). Sample characteristics for 2022 are reported in eTable 6 in Supplement 1.

**Figure 4.**
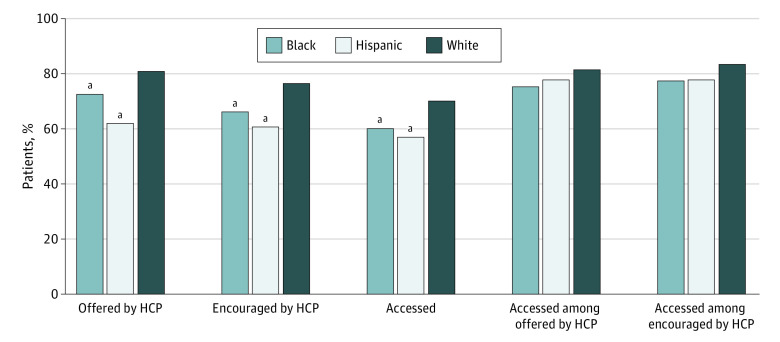
Racial and Ethnic Disparities in Patient Reports of Being Offered or Encouraged to Use a Portal by an HCP and Patient-Reported Portal Access (Overall and Among Those Who Were Offered or Encouraged) Only includes individuals who accessed their online medical record and had a health care visit in the past 12 months. Data collected from US Health Information National Trends Survey 6 (2022). HCP indicates a health care provider (ie, an individual practitioner). ^a^Statistically significant difference between outcomes and race and ethnicity, with White as the reference group to allow for comparison with prior literature. (*P* < .05). Supporting data are available in eTable 7 in Supplement 1.

In 2022, Black and Hispanic individuals reported being offered online access to their medical records by their HCP at significantly lower rates than White individuals (73% vs 81%; χ^2^_1_ = 22.24; *P* < .001 and 62% vs 81%; χ^2^_1_ = 135.57; *P* < .001, respectively). Fewer than two-thirds of Black and Hispanic individuals reported being encouraged to use an online medical record or patient portal by any HCP compared with 77% of White individuals (66% vs 77%; χ^2^_1_ = 31.35; *P* < .001; and 61% vs 77%; χ^2^_1_ = 81.30; *P* < .001, respectively). Black and Hispanic individuals also accessed a patient portal at significantly lower rates compared with White individuals (60% vs 70%; χ^2^_1_ = 23.80; *P* < .001; and 57% vs 70%; χ^2^_1_ = 49.02; *P* < .001, respectively). However, there were no significant differences in rates of portal access by race or ethnicity among those who were offered a portal or encouraged to use it (Figure 4; eTable 7 in Supplement 1).

Furthermore, there were no disparities in the use of patient portals among those who had access (Table). Conversely, Black and Hispanic individuals who accessed a portal had significantly higher rates of portal use to download health information to a computer or mobile device compared with White individuals (41% vs 28%; χ^2^_1_ = 25.66; *P* = .002; and 43% vs 28%; χ^2^_1_ = 37.56; *P* < .001). There were also no significant differences in patient-reported understanding of information among those who accessed their online medical records (Table). Most individuals (88% to 91%) who accessed their portal in 2022 indicated it was very or somewhat easy to understand the health information contained in their online medical record or patient portal.

**Table.** Patient Portal Use and Understanding of Information Among Those Who Accessed a Portal, by Race and Ethnicity, 2022

Outcome	Black (n = 1073)			Hispanic (n = 1119)			White (n = 3528)
	No. (%)^a^	χ^2^_1_	*P* value	No. (%)^a^	χ^2^_1_	*P* value	No. (%)^a^
**Patient-reported use of portal** ^b^							
Look up test results	466 (89)	0.96	.50	429 (89)	1.51	.37	1980 (91)
Download health information	202 (41)	25.66	.002	193 (43)	37.56	<.001	586 (28)
Send information to a third party	108 (24)	5.82	.15	116 (24)	5.89	.09	374 (19)
View clinical notes	381 (75)	1.02	.47	333 (67)	5.68	.15	1564 (72)
**Patient-reported understanding of information in portal** ^b^							
Very/somewhat easy	459 (90)	0.72	.47	432 (88)	4.86	.12	1943 (91)

^a^ Weighted percentage.

^b^ Only includes individuals who accessed their online medical record and had a health care visit in the past 12 months. The last 2 columns in each panel report results from a χ^2^ test of independence between outcomes and race and ethnicity, with White as the reference group to allow for comparison with prior literature.

Source: HINTS 6 (2022).

## Discussion

The findings of this study indicate that the share of individuals nationwide who were offered and accessed their online medical record has more than doubled in the past 8 years. Notably, patient access increased sharply during the COVID-19 pandemic from 46% in 2020 to 68% in 2022. This increase is likely attributable to several factors, including greater reliance on telemedicine and virtual visits during the pandemic and increased demand for access to patient portals to view test results and communicate with HCPs. Patient access also may have increased because individuals were given more opportunities to access their EHI once the health care industry began implementation of the Cures Rule, enacted to improve access, exchange, and use of EHI by patients and HCPs.

Specifically, the Cures Rule aims to improve the accessibility and ease by which individuals access and manage their EHI, eg, by using a health app of their choice.^4^ Since adoption of the Cures Rule, the share of individuals who reported being offered online access to a patient portal by an HCP and being encouraged to use it increased significantly between 2020 and 2022, which suggests that HCPs became more aware of opportunities and the importance of providing patients with access to their EHI. Rates of patient portal access were substantially higher among those who were offered a portal (81%) and encouraged to use it (83%) compared with the national average (68%), which underscores the important role that HCPs play in facilitating patient access. Furthermore, more than half of individuals (51%) who accessed their portal in 2022 did so using a health app—a 38% increase from 2020—of which, 32% used both an app and a website. This change suggests that patients had more opportunities and multiple methods of accessing their EHI in 2022. The shift toward app-based access to EHI may be attributable to health care organizations increasingly making mobile health apps available to patients, or more generally to individuals’ increased use of smartphones to manage their health information and track progress on health-related goals.^17^ Although having the option to access EHI on multiple devices may be associated with greater use, future work should assess whether there are differences in the functionality or accessibility of web-based portals vs app-based access to online medical records and how they affect use.

Despite the marked progress in overall patient access from 2014 to 2022, disparities in patient access persist. We found that in 2022 among the study sample, there were significant racial and ethnic disparities in patient reports of being offered and encouraged to use a portal by an HCP, which may have perpetuated existing disparities in patient access to EHI.^13^ Our findings suggest that growth in patient access has not occurred equitably given that only 60% of Black individuals and 57% of Hispanic individuals in this national sample accessed a patient portal in 2022 compared with 70% of White individuals. However, there were no significant differences in patient portal access among individuals who were offered a patient portal and were encouraged to use it, which suggests that disparities in patient access are likely attributable to a lack of opportunity to access the portal (ie, not being offered patient portal credentials or receiving care in settings that do not use patient portals) or the benefits of access are not being communicated. There were also no significant differences in the use or understanding of information available in the online medical records among individuals who had the opportunity to access them, which suggests that differences in digital literacy are not associated with subsequent access or use.

Taken together, these findings suggest that patient engagement with EHI would increase if we were to ensure equitable opportunities to access information. HCPs can play an important role in closing the gap in patient access and mitigating health disparities by routinely offering access to patient portals and encouraging their use. However, some HCPs—particularly those practicing in lower-resourced settings—may be less likely to use patient portals or have an electronic health record system with patient engagement capabilities.^18^ Providing equitable opportunities to access information will also involve overcoming barriers to access (eg, not having internet access or a way to access the portal) and addressing patient concerns with the privacy or security of information contained in portals. Interventions to increase access and use should ensure that both patients and HCPs have the information and resources needed to access, use, and understand the information contained in online medical records.^19^ Systematic organization-level changes (eg, universal access policies),^20^ technical improvements (eg, increasing the language accessibility of portals),^21^ and more targeted efforts to promote patient-practitioner trust may also help to ensure equitable access to online medical records and encourage their use.

### Limitations

This study had a few notable limitations. First, previous studies^13,15^ have shown that patients report several reasons for not accessing a portal when offered (eg, preferring to speak with a clinician directly or not having a way to access the portal). However, the 2022 HINTS did not include this question, and therefore, reasons for nonuse were not explored in this study. Furthermore, the HINTS survey does not capture other unobserved preferences (eg, distrust in the health care system, experiences with discrimination in health care). Second, this analysis relies on data from a cross-sectional, self-reported survey to make inferences about patient and HCP behavior, which may be subject to misinterpretation or recall bias. Namely, being offered or encouraged to use a portal by an HCP are open to interpretation by the respondent. However, interpretations of offers and encouragement are not expected to vary over time or by race and ethnicity. Finally, to help avoid possible recall bias, the sample was limited to those who had a recent health care visit (in the past year) and would have a reason to access and use their portal.

## Conclusions

In this repeated cross-sectional study of US adult respondents to the HINTS, reported access to and engagement with patient portals increased significantly from 2014 through 2022, but racial and ethnic disparities persisted in 2022. Encouragingly, there were no significant differences in use or understanding of information contained in online medical records among individuals who had the opportunity to access a portal. This finding suggests that efforts to promote equitable opportunities to access EHI would be successful in increasing patient access. Early evidence suggests that the Cures Rule may have helped enable patient and HCP engagement with EHI, when patients needed it most (ie, during the COVID-19 pandemic). Now, more work is needed to close the patient access gap and ensure that all individuals have an equal opportunity to access EHI and to make informed decisions about their health.

## Notes

Supplement 1. **eTable 1.** Survey Field Dates, Response Rates, and Sample Size **eTable 2.** Survey Questions Used to Create Key Outcome Measures **eTable 3.** Percent of individuals nationwide who were offered and accessed a patient portal, 2014-2022 **eTable 4.** Patient reports of being offered or encouraged to use a portal by their HCP and patient-reported portal access **eTable 5.** Methods of accessing online medical record or patient portal, 2020-2022 **eTable 6. **Sample Characteristics, 2022 **eTable 7.** Racial/ethnic disparities in patient reports of being offered or encouraged to use a portal by their HCP and patient-reported portal access

Supplement 2.
Data Sharing Statement
