# Supplementary material for: Progress and Persistent Disparities in Patient Access to Electronic Health Information
Source: JAMA Health Forum. 2023 Nov 10;4(11):e233883. doi: 10.1001/jamahealthforum.2023.3883 (PMC10638642; doi:10.1001/jamahealthforum.2023.3883)
Supplement: Supplement 1. — eTable 1. Survey Field Dates, Response Rates, and Sample Size eTable 2. Survey Questions Used to Create Key Outcome Measures eTable 3. Percent of individuals nationwide who were offered and accessed a patient portal, 2014-2022 eTable 4. Patient reports of being offered or encouraged to use a portal by their HCP and patient-reported portal access eTable 5. Methods of accessing online medical record or patient portal, 2020-2022 eTable 6. Sample Characteristics, 2022 eTable 7. Racial/ethnic disparities in patient reports of being offered or encouraged to use a portal by their HCP and patient-reported portal access [file jamahealthforum-e233883-s001.pdf]

Supplemental Online Content

Richwine C. Progress and Persistent Disparities in Patient Access to Electronic Health Information. *JAMA Health Forum.* Published online November 10, 2023. doi:10.1001/jamahealthforum.2023.3883

eTable 1. Survey Field Dates, Response Rates, and Sample Size

eTable 2. Survey Questions Used to Create Key Outcome Measures

eTable 3. Percent of individuals nationwide who were offered and accessed a patient portal, 2014-2022

eTable 4. Patient reports of being offered or encouraged to use a portal by their HCP and patient-reported portal access

eTable 5. Methods of accessing online medical record or patient portal, 2020-2022

eTable 6. Sample Characteristics, 2022

eTable 7. Racial/ethnic disparities in patient reports of being offered or encouraged to use a portal by their HCP and patient-reported portal access

This supplemental material has been provided by the authors to give readers additional information about their work.

© 2023 Richwine C. *JAMA Health Forum.*

eTable 1. Survey Field Dates, Response Rates, and Sample Size

HINTS Data Set	Survey Year	Field Dates	*N*	Overall response rate	Restricted *N*
HINTS 4, Cycle 4	2014	August – November 2014	3,667	34%	3,063
HINTS 5, Cycle 1	2017	January – May 2017	3,285	32%	2,777
HINTS 5, Cycle 2	2018	January – May 2018	3,504	33%	2,961
HINTS 5, Cycle 3	2019	January – May 2019	5,438	30%	4,709
HINTS 5, Cycle 4	2020	February – June 2020	3,865	37%	3,319
HINTS 6	2022	March – November 2022	6,252	28%	5,437
**Total**			26,021		22,266

Source: HINTS 4, Cycle 4 (2014) , HINTS 5, Cycles 1-4 (2017-2020), HINTS 6 (2022).
Notes*:* The HINTS Survey was not fielded in 2015, 2016, or 2021. *N* represents the overall sample size for each survey year, whereas the Restricted *N* represents the sub-sample used for analyses (i.e., respondents who had a healthcare visit in the past 12 months). See the HINTS website for response rate calculations, exact fielding dates, survey design, and weighting.

eTable 2. Survey Questions Used to Create Key Outcome Measures

Measure	Survey Year	Survey Question	Measure Description
**Offered** a portal by a health care provider	2014	Have you ever been offered access to your own personal health information online through a secure website or app by your health care provider? (Yes, No)	This measure was defined as a binary variable equal to 1 if respondents indicated "Yes" they were offered a portal and equal to 0 if they indicated "No" or "Don't know." Missing values and invalid responses were excluded from the denominator.<br><br>Notes: In 2014 (HINTS 4, Cycle 4) "Don't know" was not included as a response option. Therefore, missing values (*N* = 84, 2.28%) may include respondents who would have selected this response option.<br><br>In 2018, (HINTS 5, Cycle 2), the binary variable capturing "offered" was equal to 1 if respondents indicated they were offered a portal (Q D4) *and* it was offered by their health care provider (Q D5). The variable equals 0 if the respondent indicated "No" or "Don't know" if they were offered a portal or if the portal was not offered by a health care provider.
	2017, 2019, 2020	Have you ever been offered online access to your medical records by your health care provider? (Yes, No, Don't know)	
	2018	Have you ever been offered online access to your medical records by your health care provider or insurer? (Yes, No, Don't know)<br><br>Who offered you online access to your medical records? (Health care provider, Health insurer, Something else)	
	2022	Have you ever been offered online access to your medical records (for example, a patient portal) by your health care provider (Yes, No, Don't know)	
**Encouraged** to use portal by a health care provider	2017, 2020	Have any of your health care providers, including doctors, nurses, or office staff ever	This measure was defined as a binary variable equal to 1 if respondents indicated "Yes" they

© 2023 Richwine C. *JAMA Health Forum.*

		encouraged you to use an online medical record? (Yes, No)	were encouraged to use their portal, and equal to 0 if they indicated "No." Missing and invalid responses were excluded from the denominator.
	2022	Have any of your health care providers, including doctors, nurses, or office staff ever encouraged you to use an online medical record or patient portal? (Yes, No)	
**Accessed** online medical record or patient portal	2014	How many times did you access your personal health information on-line through a secure website or app in the last 12 months? (None, 1 to 2 times, 3 to 5 times, 6 to 9 times, 10 or more times)	This measure was transformed from a categorical variable to a binary variable equal to 1 if respondents indicated they accessed their portal at least once in the past year (i.e., '1 to 2 times' or more) and equal to 0 if they reported no access ("None") or not having a portal (in 2018 and 2022). Missing and invalid responses were excluded from the denominator.<br><br>Notes: In 2018 (HINTS 5, Cycle 2) respondents who did not report being offered a patient portal were instructed to skip questions related to portal access. These responses were coded as 0 since it is assumed that respondents who were never offered a portal were not able to access it.<br><br>In 2022 (HINTS 6), a new response option was added: "I do not have an online medical record or patient portal that was offered to me by a healthcare provider or insurer." These responses were coded as 0 since it is assumed that respondents who were never offered a portal were not able to access it.
	2017, 2018, 2019, 2020	How many times did you access your online medical record in the last 12 months? (None, 1 to 2 times, 3 to 5 times, 6 to 9 times, 10 or more times)	
	2022	How many times did you access your online medical record or patient portal in the last 12 months? (I do not have an online medical record or patient portal that was offered to me by a healthcare provider or insurer, 0, 1 to 2 times, 3 to 5 times, 6 to 9 times, 10 or more times)	
**Method of accessing** online medical record or patient portal	2020, 2022	How did you access your online medical record or patient portal? (App, Website, Both app and website, Don't know)	This measure was transformed from a categorical variable to 4 binary variables indicating "App only" access (Y/N), "Website only" access (Y/N), "Both app and website" access (Y/N), and "Don't know" (Y/N). Missing and invalid responses (including respondents who did not access or do not have a portal) were excluded from the denominators.
**Used** online medical record or patient portal	2022	In the past 12 months, have you used your online medical record or patient portal to…	This measure consists of 4 binary variables indicating use of the patient portal to "Look up test

© 2023 Richwine C. *JAMA Health Forum.*

		a. Look up test results?<br>b. Download your health information to your computer or mobile device, such as a cell phone or tablet?<br>c. Electronically send your medical information to a third party (such as another health care provider, a family member, or a smartphone health app)?<br>d. View clinical notes (a health care provider's written notes that describe your visit)?	results" (a), "Download health information (b), "Send information to a 3rd party" (c), or "View clinical notes" (d). Each variable is equal to 1 for respondents who indicated "Yes" for a given use and equal to 0 if they indicated "No." Missing and invalid responses (including respondents who did not access or do not have a portal) were excluded from the denominator.
**Ease of understanding information** in online medical record or patient portal	2022	How easy or difficult was it to understand the health information in your online medical record or patient portal? (Very easy, Somewhat easy, Somewhat difficult, Very difficult)	This measure was transformed from a categorical to a binary variable equal to 1 for respondents who indicated it was 'very' or 'somewhat' *easy* to understand information in their portal and equal to 0 for those who indicated it was 'very' or 'somewhat' *difficult*. Missing and invalid responses (including respondents who did not access or do not have a portal) were excluded from the denominators.

Source: HINTS 4, Cycle 4 (2014) , HINTS 5, Cycles 1-4 (2017-2020), HINTS 6 (2022).
Notes*:* Survey instruments are available here at the following links: HINTS 4, Cycle 4 (2014); HINTS 5, Cycle 1 (2017); HINTS 5, Cycle 2 (2018); HINTS 5, Cycle 3 (2019); HINTS 5, Cycle 4 (2020); HINTS 6 (2022).

© 2023 Richwine C. *JAMA Health Forum.*

eTable 3. Percent of individuals nationwide who were offered and accessed a patient portal, 2014-2022

	2014	2017	2018	2019	2020	2022
	No. (Weighted %)					
Offered patient portal by HCP	1,101 (37)	1,526 (52)*	1,607 (53)	2,850 (61)*	2,042 (62)	4,055 (77)*
Accessed a patient portal	869 (31)	949 (33)	1,036 (36)	2,015 (44)*	1,477 (46)	3,468 (68)*

Source: HINTS 4, Cycle 4 (2014) , HINTS 5, Cycles 1-4 (2017-2020), HINTS 6 (2022).
Notes: Only includes individuals who had a health care visit in the past 12 months.* Indicates statistically significant increase from prior year (*P* < .05). See Chi-square test output below.

Statistical Testing for Figure 1	Chi-square	*P* value
Offered patient portal by HCP		
2014 to 2017	$\chi^2(1) = 120.5$	<.001
2017 to 2018	$\chi^2(1) = 1.37$	0.51
2018 to 2019	$\chi^2(1) = 41.22$	<.001
2019 to 2020	$\chi^2(1) = 1.67$	0.48
2020 to 2022	$\chi^2(1) = 218.15$	<.001
Accessed a patient portal		
2014 to 2017	$\chi^2(1) = 3.94$	0.19
2017 to 2018	$\chi^2(1) = 4.64$	0.17
2018 to 2019	$\chi^2(1) = 53.55$	<.001
2019 to 2020	$\chi^2(1) = 3.87$	0.21
2020 to 2022	$\chi^2(1) = 392.04$	<.001

eTable 4: Patient reports of being offered or encouraged to use a portal by their HCP and patient-reported portal access (overall and among those who were offered or encouraged).

	2020	2022	Chi-square	*P* value
	No. (Weighted %)			
Offered patient portal by HCP	2,042 (62)	4,055 (77)	$\chi^2(1) = 218.15$	<.001
Encouraged to use portal by HCP	1,771 (55)	3,757 (73)	$\chi^2(1) = 319.49$	<.001
Accessed among offered by HCP	1,378 (70)	3,241 (81)	$\chi^2(1) = 110.70$	<.001
Accessed among encouraged	1,233 (70)	3,080 (83)	$\chi^2(1) = 125.21$	<.001

Source: HINTS 5, Cycle 4 (2020) and HINTS 6 (2022). Notes: Only includes individuals who had a health care visit in the past 12 months.

© 2023 Richwine C. *JAMA Health Forum.*

eTable 5: Methods of accessing online medical record or patient portal, 2020-2022

	2020	2022	Chi-square	*P* value
	No. (Weighted %)			
App	185 (16)	591 (19)	$\chi^2(1) = 4.73$	0.34
Website	865 (60)	1,728 (47)	$\chi^2(1) = 78.63$	<.001
Both app and website	291 (21)	1,056 (32)	$\chi^2(1) = 78.46$	<.001
Don't know	46 (3)	63 (2)	$\chi^2(1) = 7.07$	0.07

Source: HINTS 5, Cycle 4 (2020) and HINTS 6 (2022). Notes: Only includes individuals who accessed their online medical record and had a health care visit in the past 12 months.

© 2023 Richwine C. *JAMA Health Forum.*

eTable 6. Sample Characteristics, 2022

	No. (N = 5,437)	Weighted %
Gender Identity		
Male	1,938	46%
Female	3,160	53%
Transgender	3	0%
Gender non-conforming or other	40	1%
Race and Ethnicity		
White, Non-Hispanic	3,159	64%
Black, Non-Hispanic	936	12%
Hispanic	908	15%
Asian, Non-Hispanic	253	5%
Other, Non-Hispanic	181	4%
Age		
18-34	769	23%
35-46	870	20%
47-64	1,781	33%
65+	2,017	23%
Education		
Less than high school	324	6%
High school grad	968	21%
Some college	1,548	39%
College grad	2,597	34%
Income		
< $35,000	1,421	24%
$35,000 to $74,999	1,460	30%
> $75,000	1,980	46%
Location		
Urban	4,718	88%
Rural	719	12%
Internet Access		
Yes	4,581	88%
No	854	12%
Insurance Status		
Insured	5,080	93%
Uninsured	357	7%
Health Status		
Chronic condition	3,663	62%
No chronic condition	1,774	38%

Source: HINTS 6 (2022). Notes: Only includes individuals who had a health care visit in the past 12 months (N = 5,437).

© 2023 Richwine C. *JAMA Health Forum.*

eTable 7: Racial/ethnic disparities in patient reports of being *offered* or *encouraged* to use a portal by their HCP and patient-reported portal *access* (overall and among those who were offered or encouraged).

	Black (N = 1,073)			Hispanic (N = 1,119)			White (N = 3,528)
	No. (%)[i]	Chi-square	P value	No. (%)[i]	Chi-square	P value	No. (%)[i]
Offered by HCP	649 (73)	$\chi^2(1)$ = 22.24	<.001	556 (62)	$\chi^2(1)$ = 135.57	<.001	2,536 (81)
Encouraged by HCP	600 (66)	$\chi^2(1)$ = 31.35	<.001	522 (61)	$\chi^2(1)$ = 81.30	<.001	2,330 (77)
Accessed	519 (60)	$\chi^2(1)$ = 23.80	<.001	486 (57)	$\chi^2(1)$ = 49.02	<.001	2,165 (70)
Accessed among offered by HCP	479 (76)	$\chi^2(1)$ = 9.59	0.07	433 (78)	$\chi^2(1)$ = 3.30	0.23	2,062 (82)
Accessed among encouraged by HCP	452 (78)	$\chi^2(1)$ = 0.81	0.48	412 (78)	$\chi^2(1)$ = 17.36	0.02	1,958 (84)

Source: HINTS 6 (2022). Notes: Only includes individuals who accessed their online medical record and had a health care visit in the past 12 months. The last two columns in each panel report results from a chi-square test of independence between outcomes and race/ethnicity where White (Non-Hispanic) is the reference group. [i] Weighted percent.

© 2023 Richwine C. *JAMA Health Forum.*
