# Supplementary material for: Progress and Persistent Disparities in Patient Access to Electronic Health Information
Source: JAMA Health Forum. 2023 Nov 10;4(11):e233883. doi: 10.1001/jamahealthforum.2023.3883 (PMC10638642; doi:10.1001/jamahealthforum.2023.3883)
Supplement: Supplement 2. — Data Sharing Statement [file jamahealthforum-e233883-s002.pdf]

Data Sharing Statement

Richwine. Progress and Persistent Disparities in Patient Access to Electronic Health Information. *JAMA Health Forum*. Published November 10, 2023. doi:10.1001/jamahealthforum.2023.3883

Data
Data available: No

Additional Information
Explanation for why data not available: Data are publicly available on the Health Information National Trends Survey website: https://hints.cancer.gov/data/download-data.aspx
